# Food Fights and Femur Wars

**DOI:** 10.1100/tsw.2001.8

**Published:** 2001-02-23

## Abstract

Nature leads this week with a story about new rules for planting genetically modified (GM) seeds in Europe. Science chose to lead off the week with a debate about the oldest ancestor of modern day humans.

Biotechnologists, farmers, and the European Union (EU) moved a step closer to permitting the use of transgenic crops throughout Europe, reports *Nature* in their lead story. At present, public outcry over the so-called frankenfoods has forced the 15 EU member states to impose a *de facto* moratorium on all GM foods, says *Nature*. The new rules attempt to address these concerns on three fronts. First, all transgenic crops must undergo a "strict" assessment and be continually monitored once planted. Second, local authorities and the public will be told where, when, and why GM crops are being used in or around their communities. Third, as of year 2004 GM plants containing antibiotic-resistant genes could no longer be sold to consumers, reports *Nature*, and the rules forbid the experimental use of antibiotic resistant GM organisms after 2008. Biotechnologists and officials in nine EU states hailed the rules and hoped to soon lift the ban on transgenic crops. Six EU members - France, Italy, Greece, Denmark, Austria and Luxembourg - rejected the rules and intend to block GM crops. Environmentalists split the difference, sort of. They support the rules as long as the ban remains in place, reports *Nature*. *Science* did not cover this story.

*Science* leads off with the uproar caused by the discovery of 6-million-year-old teeth and leg bones in Kenya. The team that discovered scattered teeth, a jaw bone, and pieces of femurs of the "Millenium Ancestor," so named because the bones were unearthed in 2000, claims that these particular fragments are from the earliest hominid ancestor of the human race. Their argument is partly based on the way the leg bone connects to the hip bone. The femoral head of one leg bone is much larger than that of another human ancestor, the famous Lucy skeleton, reports *Science*. Large femur heads usually imply that the species could walk on two legs. Others disagree. They argue that the femur heads collected from male members of Lucy's species are much larger than Millenium Ancestor's, says *Science*. These scientists doubt that the Millenium Ancestor is a distant relative of ours, reports *Science*. Many suspect that this creature either evolved into an ape or became extinct. But all the scientists are reserving their final judgement until the peer-reviewed paper containing the results is released in the 28 February issue of the *Comptes Rendus de l'Académie des Sciences*. *Nature* did not cover this story.

Both *Science* and Nature ran articles about the Bush Administration's soon-to-be-officially-announced science budget. *Science* placed it in the third slot; *Nature* put it in fourth. The National Institutes of Health (NIH) was the big winner. They are expecting a 15 percent budget boost that would add $3 billion to NIH coffers. But the windfall comes at a cost that has even NIH supporters concerned. In the Bush proposal, budgets for agencies that fund the physical sciences, primarily the National Science Foundation, the Department of Energy, NASA, and the U.S. Geological Survey - will stay flat or decline to make room from Bush's promised tax cut.
